# Optimization of heroin conjugate vaccine performance in rodent models

**DOI:** 10.1038/s41541-026-01517-6

**Published:** 2026-07-14

**Authors:** Essie Komla, Connor Whalen, Oscar B. Torres, Agnieszka Sulima, Arthur E. Jacobson, Kenner C. Rice, Gary R. Matyas

**Affiliations:** 1 Laboratory of Adjuvant and Antigen Research, US Military HIV Research Program, Center for Military Infectious Diseases Research, Walter Reed Army Institute of Research, Silver Spring, MD USA; 2 Henry M. Jackson Foundation for the Advancement of Military Medicine, Bethesda, MD USA; 3 Drug Design and Synthesis Section, Molecular Targets and Medications Discovery Branch, Intramural Research Program, National Institute on Drug Abuse and the National Institute on Alcohol Abuse and Alcoholism, National Institutes of Health, Department of Health and Human Services, Bethesda, MD USA; 4 Present Address: bioMerieux, 1201 S 4800 W, Salt Lake City, UT USA; 5 Present Address: Merck Sharp & Dohme LLC., Rahway, NJ USA

**Keywords:** Drug discovery, Immunology, Medical research, Neuroscience

## Abstract

Opioid use disorder (OUD) remains a global public health concern, exerting a significant economic burden on societies worldwide. To combat the rise of substance use disorder, a complementary vaccine-based intervention was developed to support and strengthen the existing treatment modalities. Hapten-protein conjugate vaccines are employed as a promising novel strategy for neutralizing nonimmunogenic small-molecule drugs such as opioids by eliciting drug-specific antibodies that sequester opioids in circulation before reaching the brain. In this study, multiple parameters to optimize the efficacy of the TT-6-AmHap heroin conjugate vaccine were investigated in mice and rats, focusing on dose optimization, hapten density, booster timing, and cross-reactivity with other prescription opioids. Binding antibody responses were quantified, and vaccine efficacy was assessed using nociceptive assays following a drug challenge. Immunization with TT-6-AmHap vaccine in combination with adjuvants ALF43 and Alhydrogel^®^ (ALFA), demonstrated a robust in-vivo efficacy across multiple opioid challenges including heroin, hydrocodone, and hydromorphone. Both antibody titers to 6-AmHap and protective efficacy were maintained following repeated heroin challenges up to 54 weeks. Notably, these repeat heroin challenges did not induce any measurable decline in antibody titers. Further characterization of the vaccine responses indicated a strong correlation between hapten density on the carrier protein and both binding antibody titers and overall vaccine efficacy. These findings support the continued development of hapten-based conjugate vaccines for OUD and demonstrate that an optimized vaccine can provide long-lasting protection against the antinociceptive effects of opioids in animals, laying the groundwork for future translational studies.

## Introduction

The opioid crisis continues to pose a significant public health challenge, with opioid use disorder (OUD) contributing to rising rates of addiction, overdose, and mortality worldwide. While the World Health Organization (WHO) estimated that 316 million people worldwide used drugs in 2023, the 61 million engaged in non-medical opioid use accounted for a staggering 75% of all drug-related deaths^1^. This trend was acutely mirrored within the European Union (EU). According to the European Drug Report 2025, although opioid use was limited to approximately 0.3% of the adult population (860,000 individuals), the lethality of these substances was profound^2^. In 2023 alone, the EU recorded 7,459 overdose deaths, a rate of 24.7 per million, with heroin identified in 70% of all fatal cases^3^. In the United States, 8.9 million Americans have reported misusing opioids over a 12-month span, with 660,000 of them specifically abusing heroin in 2023, according to the National Center for Drug Abuse Statistics^4^. The overall drug overdose deaths in the U.S. have dropped by roughly 27% in 2024 compared to 2023, marking the lowest rate recorded since 2019^5,6^. Despite the progressive decline in recent years, heroin-related overdose deaths continue to claim more than 2,500 lives each year, with many cases involving synthetic opioids^6^. The economic burden of opioid misuse in the U.S., including heroin is staggering, with estimates reaching $1.5 trillion in 2020, driven by healthcare costs, loss of productivity, and legal programs^7,8^. These statistics underscore the urgent need for innovative therapeutic strategies to mitigate the impact of drugs of abuse.

While medication-assisted therapies (MAT) such as methadone, buprenorphine, and naltrexone have shown efficacy in treating OUD, these approaches are often limited by issues of adherence, accessibility, and side effects^9^. A promising addition to traditional drug-based treatments is immunopharmacotherapy, in which through vaccination, antibodies are induced to sequester exogenous opiates before they can penetrate the blood-brain barrier and act on the opioid receptors in the brain^10–12^. Unlike traditional vaccines that work by targeting disease-causing organisms, opioid vaccines function by coupling a small-molecule drug analog (hapten) to a larger immunogenic carrier protein. This enables the immune system to recognize and mount a response against otherwise non-immunogenic substances. Upon immunization, the host generates high-affinity antibodies that bind the target drug in circulation, forming immune complexes that are trapped in the bloodstream, thus preventing the drug from exerting its psychoactive effects on the central nervous system.

Three major components of opioid vaccines include a drug hapten, a carrier protein, and an adjuvant, which work in tandem to elicit a strong hapten-specific response. Specifically, haptens such as FEN-CRM for fentanyl or Oxy(Gly)4 for oxycodone are conjugated to large carrier proteins like CRM197 (diphtheria toxin mutant) or sKLH (succinylated Keyhole Limpet Hemocyanin), and paired with adjuvants such as the dmLT, or aluminum, respectively to maximize the production of drug-specific antibodies. Preclinical findings have demonstrated remarkable efficacy for these compositions, with studies in rodent models showing a significant reduction in opioid-induced antinociception, respiratory depression, and bradycardia, as well as an attenuated drug-seeking behavior and self-administration, and a decreased opioid brain distribution^13–17^. The TT-6-AmHap heroin conjugate vaccine developed in our laboratory combines a heroin-derived hapten (6-AmHap) with tetanus toxoid (TT) and adjuvants such as ALF43 and Alhydrogel^®^ (ALFA), eliciting high-affinity IgG specific antibodies against heroin and its metabolites^18^. Previous studies have shown that TT-6-AmHap significantly reduced heroin-induced antinociception in rodents and maintained durable immune protection for at least six months^18^.

Despite the well-established concept of opioid vaccines, enhancing their efficacy and translational potential requires further optimization of several critical design factors. These include the optimal vaccine dose, the number of haptens conjugated per carrier protein, the timing of booster dose administration, and the breadth of protection against structurally related opioids. These variables are essential for achieving a robust and long-lasting antibody response. Moreover, the relationship between immunogenicity and behavioral outcomes, particularly across different species and behavioral assays requires further evaluation. In this study, we systematically evaluated the effects of TT-6-AmHap vaccine dose, hapten density, and booster timing on antibody production and behavioral protection in mice and rats. We also assessed the durability of the antibody response and the vaccine’s cross-reactivity with other drugs of abuse. Behavioral efficacy was measured using the tail-immersion, hot plate, and thermal place preference tests following a drug challenge. Our findings provide crucial insights into the rational design and optimization of opioid conjugate vaccines and support their continued development as a complimentary therapeutic strategy for OUD.

## Results

**Fig. 1:**
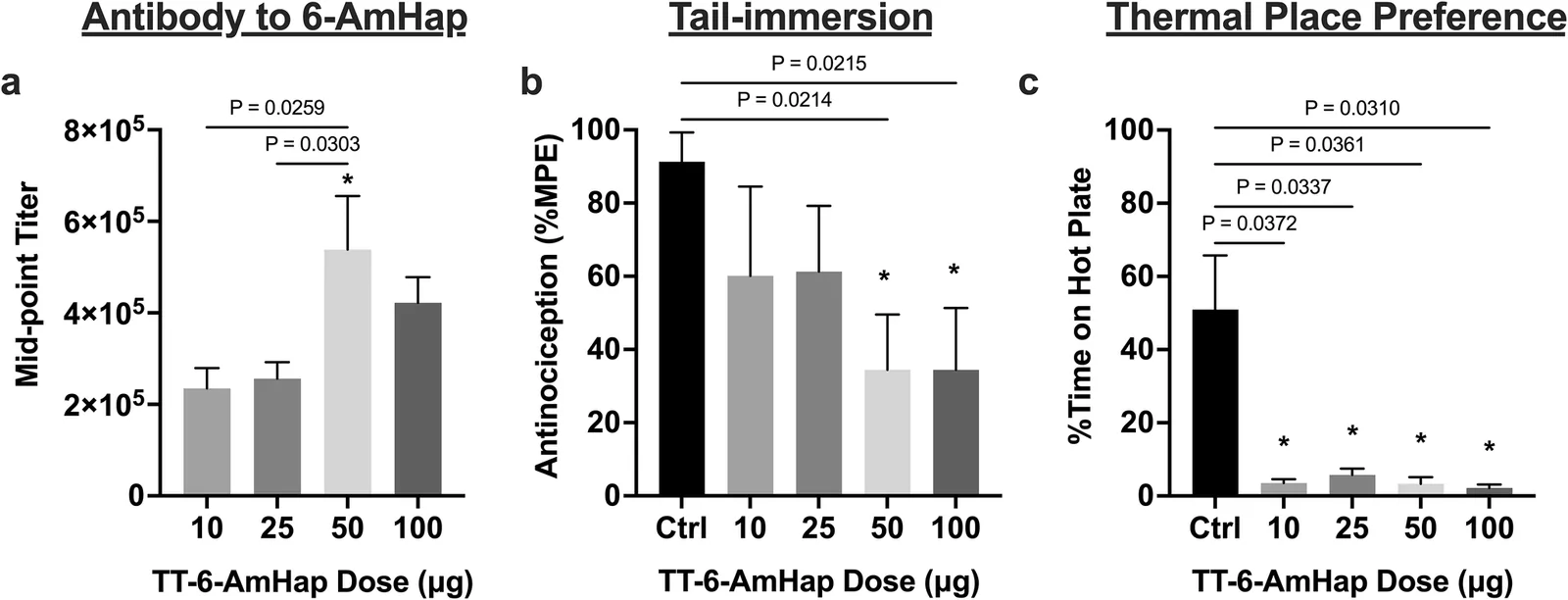
Comparison of TT-6-AmHap doses for serum IgG specific response and efficacy against heroin challenge in rats. Rats were immunized with increasing doses of TT-6-AmHap at weeks 0, 3, 6, and 14. **a** 6-AmHap specific antibodies were determined by ELISA at week 16. Vaccine efficacy was assessed by (**b**) tail-immersion and **c** thermal place preference tests at week 18 after a heroin challenge dose administration. Statistical differences between groups were determined by one-way ANOVA with Dunnett’s multiple comparisons test. For the ELISA data, values represent the mean ± SEM of triplicate determinations; *N* = 5–6/group; *p* < 0.05 compared to 10 and 25 μg TT-6-AmHap. For the tail-immersion and thermal place preference tests values represent the mean ± SEM; *N* = 5–10/group; *p* < 0.05 compared to control group (Ctrl). No star symbol indicates not significant (ns).

To determine the optimal TT-6-AmHap dose that will induce high antibody titers and protect against heroin-induced antinociception in animals, male rats were immunized with increasing doses of the TT-6-AmHap vaccine in combination with liposomal adjuvant, Army Liposome Formulation with 43% cholesterol (ALF43) and Alhydrogel^®^ (ALFA). The animals were immunized with four doses of TT-6-AmHap (10 µg, 25 µg, 50 µg, or 100 µg) at weeks 0, 3, 6, and 14. At week 16, serum 6-AmHap specific antibody titers were determined in all animals. Immunization with any of the TT-6-AmHap doses induced high antibody titers to 6-AmHap (Fig. 1a). Although the 10 µg and 25 µg doses were not significantly different from one another, increasing the dose to 50 µg TT-6-AmHap significantly increased the mid-point titers from 234,748 (for 10 µg) and 256,414 (for 25 µg) to 538,502. Further increasing the dose to 100 µg, decreased the mid-point titer to 422,130, but this value was not significant.

At week 18, TT-6-AmHap vaccine efficacy was assessed in the rats following a heroin challenge dose administration by the use of nociceptive assays. The animals were challenged with 0.5 mg/kg heroin via intravenous (IV) tail injection and tested in the tail-immersion (TI) and thermal place preference (TPP) assays 20 min post the challenge dose administration. The vaccine induced a significantly lower heroin-induced antinociception at the 50 µg and 100 µg TT-6-AmHap doses in the TI assay and at all the doses tested in the TPP test compared to the controls (Fig. 1b, c). The reduced heroin-induced antinociception in the animals indicated the efficacy of the TT-6-AmHap vaccine at the doses tested.

**Fig. 2:**
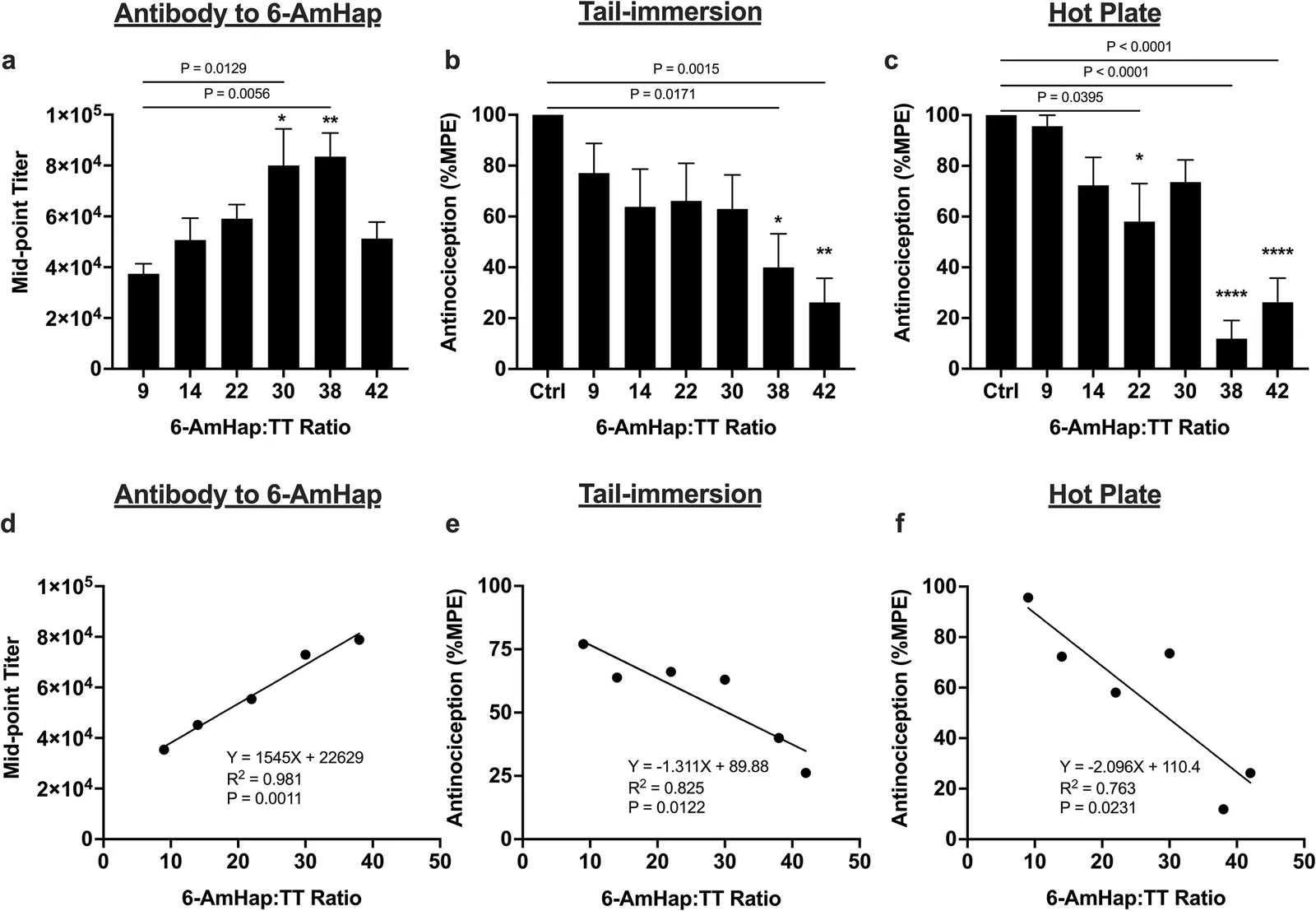
TT-6-AmHap response to varying hapten density immunization in mice. Animals were immunized with increasing molar ratio of 6-AmHap to tetanus toxoid. **a**, **d** 6-AmHap serum specific IgG response was determined at week 16 by ELISA. Antinociceptive response to a heroin challenge at week 18 was assessed by (**b**, **e**) tail-immersion and (**c**, **f**) hot plate assays. Statistical differences between groups were determined by one-way ANOVA with Tukey’s multiple comparisons test. For the ELISA data, values represent the mean ± SEM of triplicate determinations; *N* = 10/group; *p* < 0.05 compared to 9 6-AmHap:TT ratio. For the tail-immersion and hot plate assays, values represent the mean ± SEM; *N* = 10/group; *p* < 0.05 compared to control group (Ctrl). No star symbol indicates not significant (ns). Linear correlation function was used for (**d**, **e**, **f**) data analysis.

To determine the optimal number of 6-AmHap haptens required to elicit the maximal antibody titers and protect against heroin-induced antinociception in animals, female mice were immunized with 10 µg TT-6-AmHap containing varying hapten densities ranging from 9 to 42 6-AmHap haptens conjugated to tetanus toxoid (TT) via protein linkers. 16 weeks post primary immunization, the mid-point titers were significantly higher in mice vaccinated with 30 and 38 6-AmHap haptens per TT molecule compared to those vaccinated with 9 (Fig. 2a). There were no significant differences between the 30 and 38 6-AmHap haptens and any other hapten numbers. At week 18, the mice were challenged with 1 mg/kg heroin via subcutaneous (SC) injection and tested in the TI and hot plate (HP) assays 15 min after heroin challenge. Heroin-induced antinociception was significantly reduced in the animals vaccinated with 38 and 42 6-AmHap haptens to TT ratio in both assays and with 22 6-AmHap haptens to TT ratio in the HP assay compared to the control mice (Fig. 2b, c). Furthermore, the correlation between antibody titers and hapten density was established, indicating a positive correlative relationship between hapten density and antibody response (Fig. 2d). Inversely, the inhibition of heroin-induced antinociception negatively correlated with hapten density, indicating that high hapten density is required for the TT-6-AmHap vaccine efficacy (Fig. 2e, f).

Following TT-6-AmHap dose optimization in rats, both 50 µg and 100 µg TT-6-AmHap doses were effective against the inhibition of heroin-induced antinociception in the TI and TPP tests (Fig. 1b, c). However, the 100 µg TT-6-AmHap dose was selected for all subsequent rat experiments to better reflect the proposed human dose to be used in a phase 1 clinical trial. The 10 µg TT-6-AmHap mice dose was established previously^19^. Pertaining to the hapten density optimization, 38 and 42 6-AmHap haptens were optimal at inducing high antibody titers and reducing heroin-induced antinociception (Fig. 2a–c) in mice. However, due to the possible steric hindrance of 42 haptens per TT molecule, 38 haptens was selected and established as the foundation for all forthcoming TT-6-AmHap vaccine formulations.

**Fig. 3:**
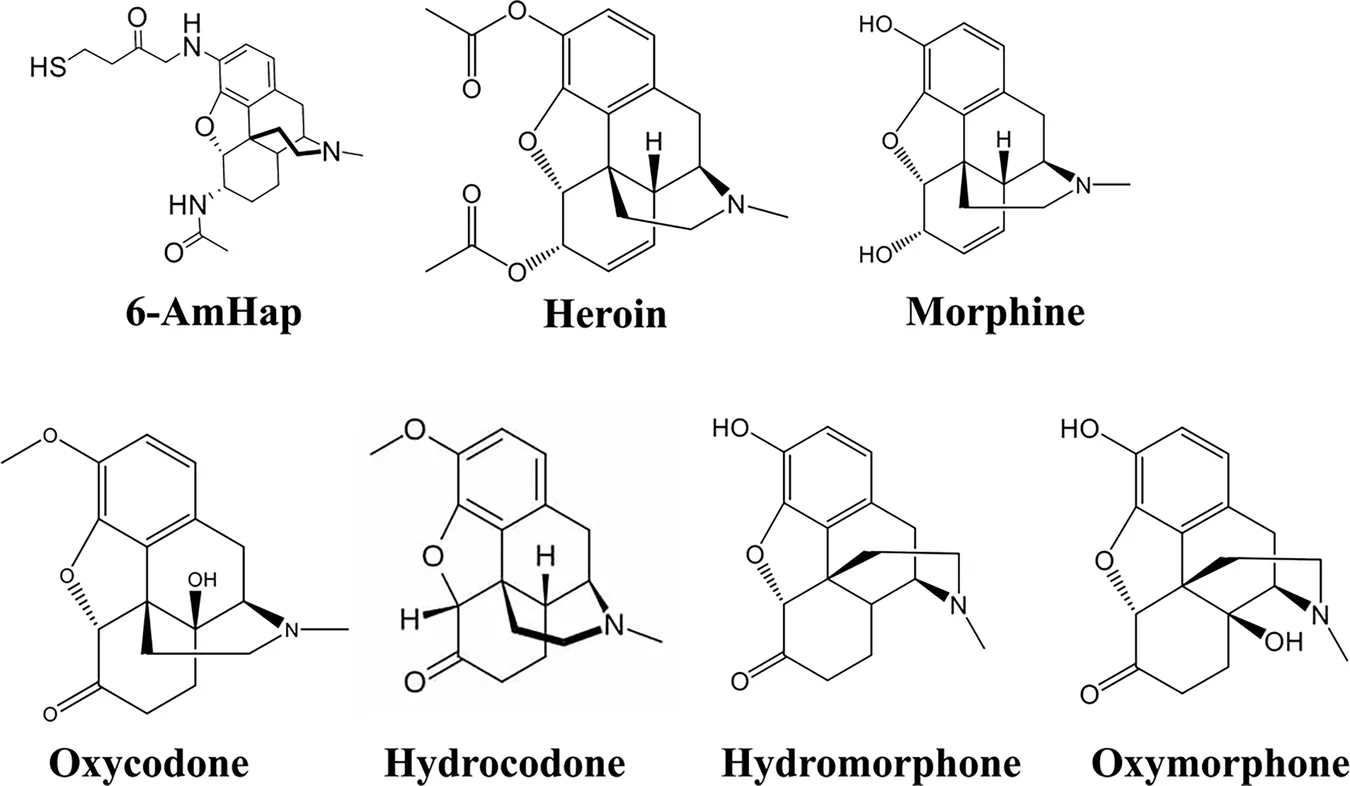
Structures of heroin hapten, 6-AmHap, heroin and other prescription opioids. Oxycodone and Oxymorphone contain a hydroxyl and the C14 position which may be responsible for lack of efficacy against these opioids.

**Fig. 4:**
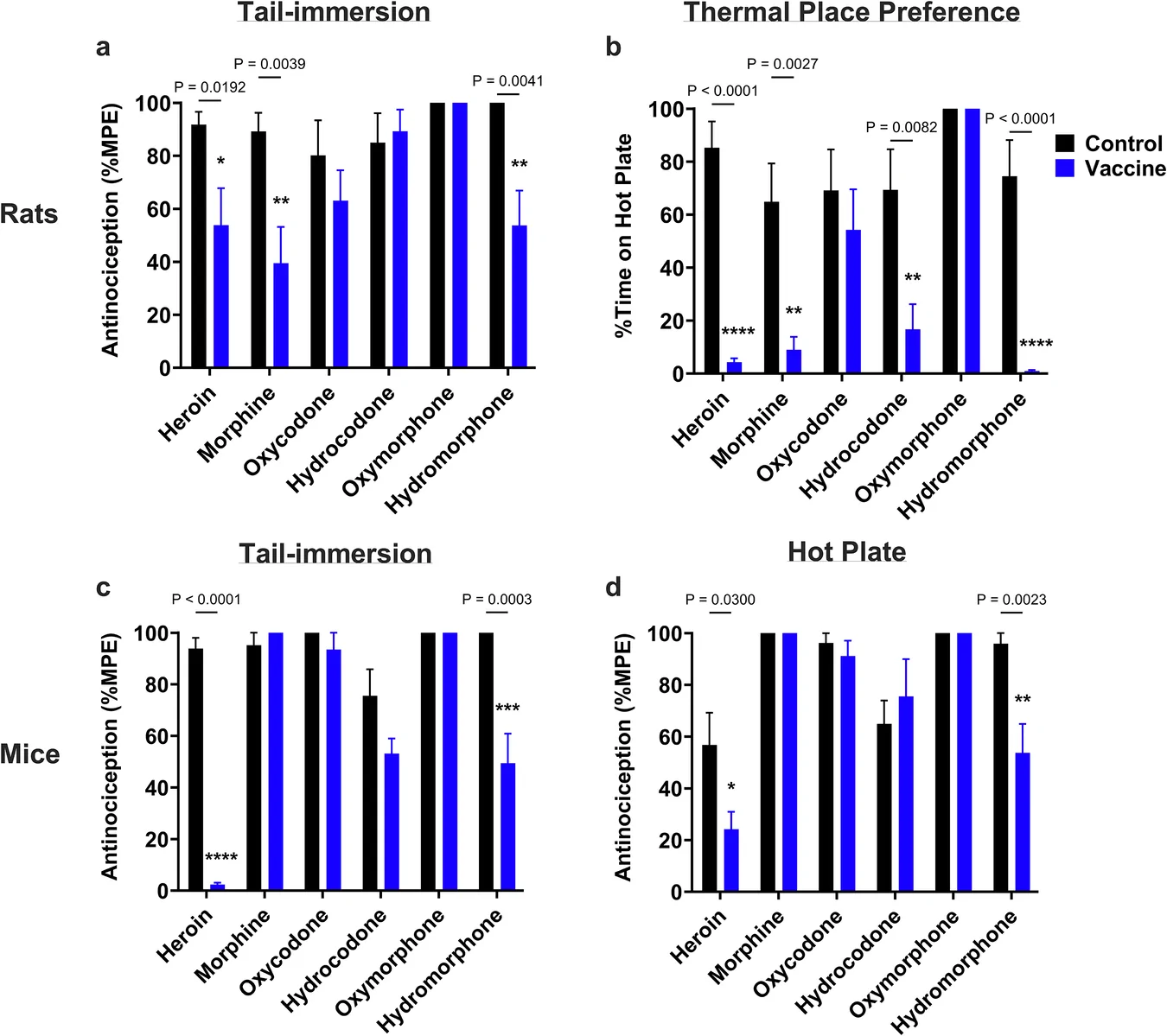
TT-6-AmHap efficacy against various opioids. Immunized rats (top panel) and mice (bottom panel) were challenged at week 18 with heroin, morphine, oxycodone, hydrocodone, oxymorphone, or hydromorphone. Antinociceptive response to the challenge dose was evaluated by (**a**, **c**) tail-immersion assay in rats and mice, (**b**) thermal place preference test in rats, and **d** hot plate assay in mice. Statistical differences between the vaccinated group and its corresponding control were determined by multiple unpaired t-tests. Data represent the mean ± SEM; *p* < 0.05; *N* = 7–10/group. No star symbol indicates not significant (ns). Note: Some animals died during the experiment due to causes not related to opioid challenge or other study procedures.

To evaluate the efficacy of the TT-6-AmHap vaccine against other prescription opioids (Fig. 3), a new batch of rats and mice were immunized with TT-6-AmHap at weeks 0, 3, 6, and 14. At week 18 (4 weeks after the last immunization), rats were challenged by the IV route with heroin (0.5 mg/kg), morphine (3.5 mg/kg), oxycodone (3 mg/kg), hydrocodone (3 mg/kg), oxymorphone (0.5 mg/kg), or hydromorphone (0.5 mg/kg). Whereas mice were challenged by the SC route with heroin (1 mg/kg), morphine (15 mg/kg), oxycodone (4 mg/kg), hydrocodone (4 mg/kg), oxymorphone (1 mg/kg), or hydromorphone (1 mg/kg). The challenge doses were selected based on a preliminary dose-response studies (Suppl. Figs. 1 and 2). Immunization with TT-6-AmHap significantly protected rats from challenge with heroin, morphine, and hydromorphone in both the TI and TPP tests and from hydrocodone in the TPP test (Fig. 4a, b). However, protection was not observed for oxycodone and oxymorphone in either of the assays (Fig. 4a, b). Similar to the rats, immunization with TT-6-AmHap significantly protected mice from heroin and hydromorphone challenge in both TI and HP assays (Fig. 4c, d). In contrast to the observation made in rats, mice were not protected from morphine challenge in either assay (Fig. 4c, d). Additionally, there was no statistical difference between immunized and control animals following challenge with oxycodone, hydrocodone, and oxymorphone in either the TI or HP assays (Fig. 4c, d).

**Fig. 5:**
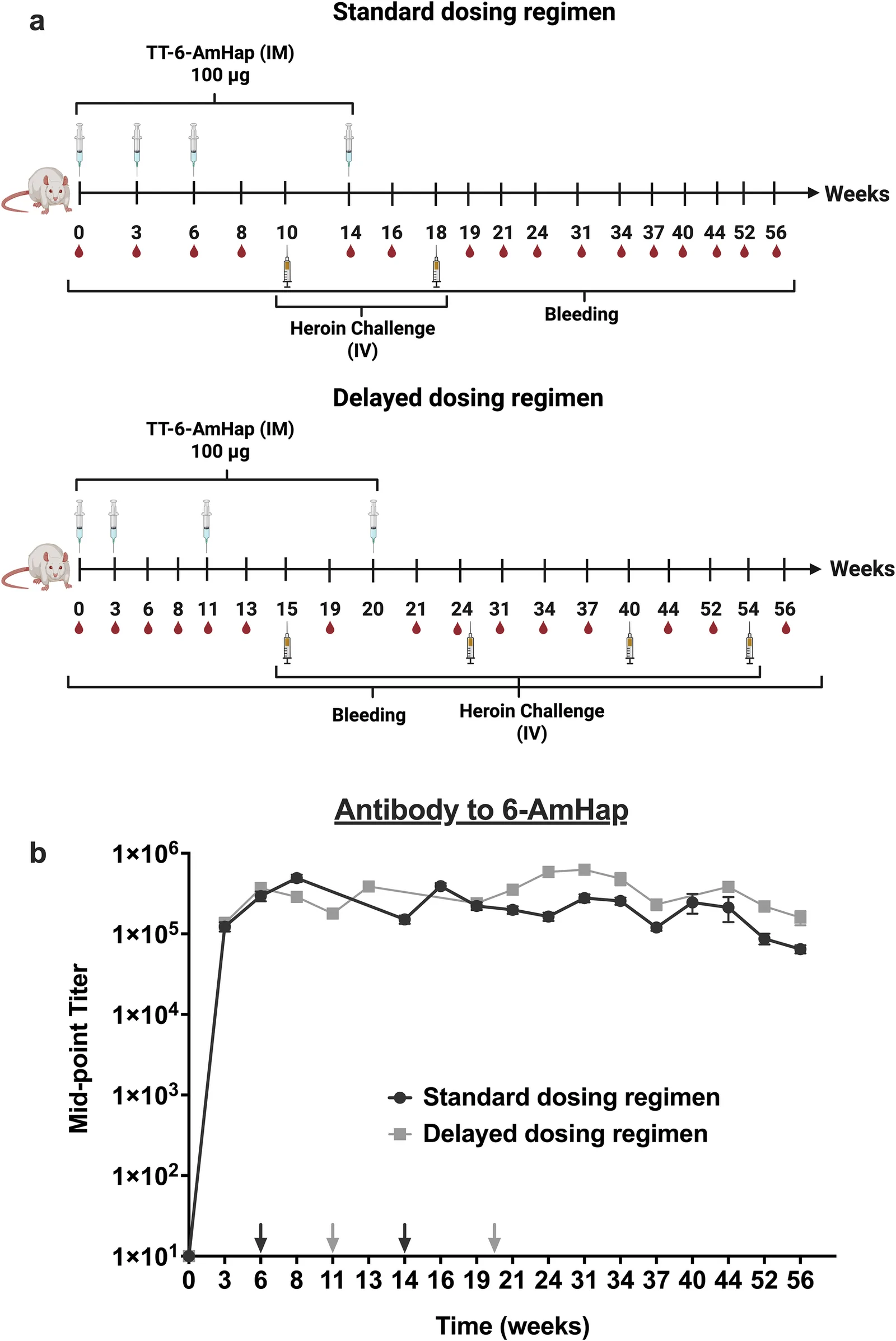
Time course of TT-6-AmHap hapten specific serum IgG antibody titers in rats. **a** Immunization protocol of the standard dosing regimen versus the delayed dosing regimen created in https://BioRender.com. **b** Serum 6-AmHap specific IgG response over a 56-week study period in rats immunized with standard dosing (dark gray circle) or delayed dosing (light gray square) regimen. Black arrows indicate standard booster dose administration at weeks 6 and 14. Light gray arrows indicate delayed booster dose administration at weeks 11 and 20. Statistical comparison between the standard dosing and delayed dosing regimen groups at a given time point used a mixed-effects analysis with Geisser-Greenhouse correction and Tukey’s multiple comparisons test. Values represent the mean ± SEM; *N* = 7–10/group. Note: Some animals died during the experiment due to causes not related to heroin challenge or other study procedures.

Delayed vaccine boosts have shown to increase the efficacy of a malaria vaccine in humans^20–23^. With this in mind, the next study was designed to test if a delay in administering a booster TT-6-AmHap immunization would improve the antibody response to 6 AmHap, to determine the impact of a heroin challenge on antibody titer, and to test the durability of the antibody response. Rats were immunized by the standard schedule at weeks 0, 3, 6, and 14 compared to a delayed immunization schedule at weeks 0, 3, 11, and 20 (Fig. 5a). Maximal antibody titers were observed after the initial TT-6-AmHap vaccine dose and remained relatively consistent for the 56-week study period in animals immunized using either the standard or delayed dosing regimen (Fig. 5b). However, analyses of both dosing curves as a whole indicates that the groups are significantly different (*p* = 0.0054) (Fig. 5b, Suppl. Table 1). Although the mid-point titers had relatively minor fluctuations over time within each group, not all of the differences were significant (Supplementary Table 2).

**Fig. 6:**
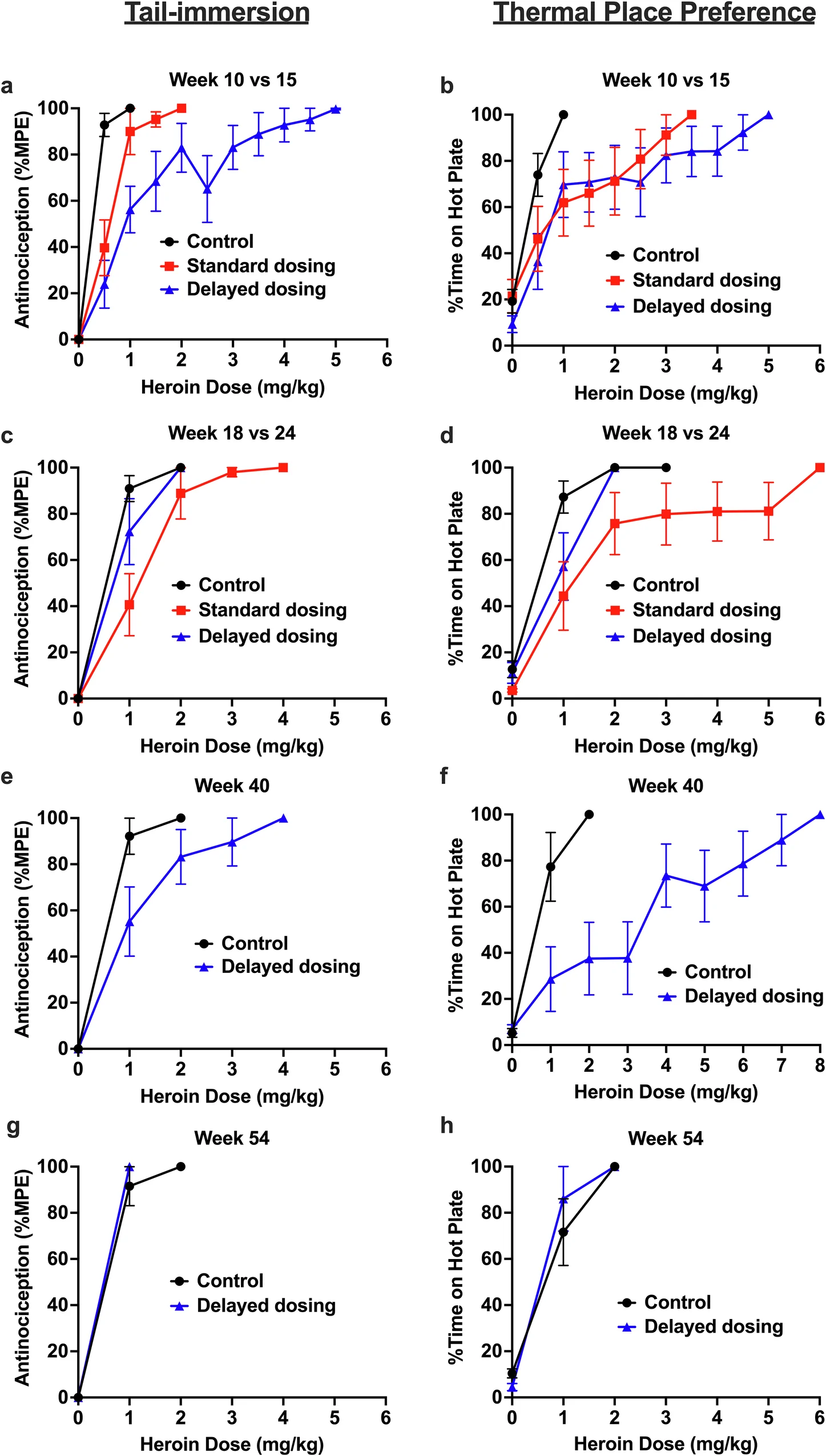
TT-6-AmHap efficacy against repeated heroin challenges in rats following a standard and delayed dosing regimen. Rats were immunized with TT-6-AmHap using the standard dosing regimen (red square) or delayed dosing regimen (blue triangle). Animals vaccinated with the standard dosing regimen were challenged with repeated heroin at weeks 10 and 18. Animals vaccinated with the delayed dosing regimen were challenged with repeat heroin at weeks 15, 24, 40, and 54. Antinociceptive response to the challenge dose was evaluated by (**a**, **c**, **e**, **g**) tail-immersion and (**b**, **d**, **f**, **h**) thermal place preference tests. Values represent the mean ± SEM; *N* = 7–10/group. Note: Some animals died during the experiment due to causes not related to heroin challenge or other study procedures.

Animals assigned to the standard dosing regimen were challenged with heroin at weeks 10 and 18, and those on the delayed regimen at weeks 15 and 24, with each challenge occurring four weeks after the third and fourth immunizations in both protocols. Animals were challenged with repeated 0.5 mg/kg heroin doses every 20 min until achieving 100% response in the TI and TPP assays at weeks 10 and 15, and with repeated 1 mg/kg heroin doses at weeks 18 and 24. Comparison of the dose-response curves at weeks 10 and 15, four weeks after the third immunization using both dosing regimens (standard vs delayed), indicated a significant difference in the dose-response curves of the vaccinated rats in the TI assay (Table 1, Fig. 6a) but not in the TPP test (Table 1, Fig. 6b). Similarly, four weeks after the fourth immunization, at weeks 18 and 24, the TI heroin dose curves were statistically different (Table 1, Fig. 6c), but not the TPP dose curves (Table 1, Fig. 6d). Moreover, the vaccinated animal dose-response curves significantly shifted from the control animal dose curves in both assays (Table 1, Fig. 6a–d).

**Table 1:** EC_50_ Values and statistical analyses of heroin repeat-dose challenges

Study Week	Assay	EC_50_^a^ (mg/kg)			Control vs Standard *p*-value	Control vs Delayed *p*-value	Standard vs Delayed *p*-value
		Control	Standard	Delayed			
10 vs. 15	TI	0.08	0.56	1.07	0.0003	< 0.0001	0.0012
10 vs. 15	TPP	0.42	0.88^b^	1.44	0.0042	0.0141	0.28
18 vs. 24	TI	0.19	1.13	0.76	0.0083	0.042	0.035
18 vs. 24	TPP	0.94	1.09	1.05	0.0031	0.0292	0.08
40	TI	0.18	ND	1.01	ND	0.0085	ND
40	TPP	0.68	ND	3.29^b^	ND	0.0006	ND
54	TI	0.72 ^b^	ND	0.50 ^b^	ND	> 0.9999	ND
54	TPP	0.76 ^b^	ND	0.72 ^b^	ND	0.688	ND

EC_50_ = half maximal effective concentration; ND = not determined.

^a^EC_50_ was not able to be reliably calculated for control animals in either the TPP or TI assays.

^b^EC_50_ values were calculated from linear regression analysis.

Since animals assigned to both dosing regimens showed similar antibody titer levels (Fig. 5b) and TPP test results (Fig. 6b, d), animals that received the standard dosing regimen were monitored without further heroin challenge. However, those on the delayed dosing regimen underwent a heroin challenge twice more at weeks 40 and 54 with 1 mg/kg heroin to assess the impact of repeated heroin exposure on antibody titers. Efficacy of the vaccine in this group compared to the naïve control group was observed in both TI and TPP assays. The immunized animals were significantly protected from heroin challenge compared to the control rats as assessed by both assays until the week 54 challenge, where protection was no longer observed (Fig. 6e–h). This indicates the vaccine is effective 20 weeks after the last immunization and after heroin challenge. In contrast, the protective efficacy had waned (week 54) 14 weeks after the week 40 challenge.

## Discussion

In this study, the antibody responses to immunization withTT-6-AmHap heroin conjugate vaccine adjuvanted with ALF43 and Alhydrogel^®^ (ALFA) and the resulting efficacy from heroin challenge was investigated in mice and rats. Vaccinated animals developed robust anti-6-AmHap antibody responses following immunization and exhibited attenuated drug-induced antinociceptive responses in the nociceptive assays following a challenge dose administration, confirming the efficacy of the vaccine. We first evaluated a dose-dependent relationship between TT-6-AmHap vaccine administration and both antibody response and protection against heroin-induced antinociception. Antibody titers increased with dose, with the 50 µg TT-6-AmHap group showing more than double the titers of the 10 µg and 25 µg TT-6-AmHap groups (Fig. 1a). Interestingly, while the 100 µg TT-6-AmHap dose also resulted in a doubling of titers compared to the lower doses, this increase was not statistically significant, suggesting a potential plateau in immunogenicity beyond the 50 µg dose. Other studies have shown similar plateauing of the antibody^24–26^. Since both the 50 and 100 µg doses had equivalent efficacy from heroin challenge in both the tail immersion and thermal place preference assays and these studies were being conducted as part of the non-clinical section of a FDA Investigation New Drug application in which 100 µg was the proposed human dose, the 100 µg dose of TT-6-AmHap was selected for further studies in the rat. Similar rat studies have also used a 100 µg antigen dose for substance abuse vaccine studies^24,25^.

Antinociceptive effects following heroin challenge revealed nuanced differences between the tail-immersion and thermal place preference tests. In the tail-immersion (TI) assay, which measures acute spinal reflexes to thermal pain, only the 50 and 100 µg groups showed significant protection against heroin challenge (Fig. 1b), indicating that higher antibody levels may be necessary to block heroin’s effects in this model. In contrast, the thermal place preference (TPP) test, which assesses the supraspinal component of thermal sensitivity, showed significant protection across all dose groups. This suggests that even lower antibody titers may be sufficient to block heroin’s impact on thermal preference behavior, potentially due to differences in central versus peripheral drug action or assay sensitivity. These results align with previous work demonstrating that anti-opioid vaccines must achieve sufficient antibody titers to sequester drug molecules in circulation and block their entry into the brain^24–28^. Despite the lack of statistical significance between the 50 and 100 µg doses in antibody titers and in behavioral assays, we selected the 100 µg TT-6-AmHap dose for future rat studies to match the dose proposed for a human phase 1 clinical trial. Additionally, this dose was tested in the rabbit pharmacology-toxicology studies and was determined to be safe^29^.

To assess the breadth of vaccine protection, we tested the efficacy of the TT-6-AmHap vaccine against other opioids. Interestingly, the vaccine failed to protect mice against morphine, oxycodone, hydrocodone, and oxymorphone-induced antinociception in both the TI and hot plate (HP) tests, despite its efficacy against heroin and hydromorphone (Fig. 4c, d). In contrast, rats showed significant protection against morphine in both the TI and TPP assays (Fig. 4a, b). This may be due to species-specific differences, sex differences, or the route of administration, and may reflect variations in opioid metabolism, immune processing, or behavioral sensitivity^30–35^. On another note, these data also highlight the importance of structural specificity in hapten design; even small molecular differences between heroin and morphine may affect antibody binding affinity and neutralization capacity^28^. Furthermore, the lack of TT-6-AmHap vaccine protection against oxymorphone and oxycodone suggests that the induced antibodies cannot tightly bind to these molecules which have a hydroxyl added to the C14 position of the morphine ring^36,37^ (Fig. 3). Consistent with our prior research, the inhibitory concentration 50% (IC₅₀) values obtained via competition ELISA demonstrated that 6-AmHap antibodies exhibit cross-reactivity with a range of opioids^18^. These findings underscore the need for tailored hapten designs when targeting multiple opioids and suggest that vaccine efficacy may vary across species and drug targets.

We also examined whether delaying the booster dose of the TT-6-AmHap vaccine would enhance antibody production and behavioral protection in rats. Both the standard and delayed dosing regimens produced relatively comparable durable antibody responses, but the delayed dosing regimen had significantly higher antibody titers. Both regimens had very high antibody titers of long duration, demonstrating that the TT-6-AmHap vaccine formulation is capable of inducing long-lasting humoral immunity, regardless of booster timing. Behaviorally, differences emerged in the TI assay, where heroin-induced antinociception varied between the two dosing regimens, but not in the TPP test. These results indicate that TT-6-AmHap vaccine efficacy may be limited to the reflexive nociceptive pathways as observed in the TI assay rather than voluntary behavioral responses to thermal stimuli as seen in the TPP test. Therefore, the TPP test represents a more rigorous test for the assessment of prevention of heroin from entering the brain. Although the antibody titer was durable with high mid-point titers throughout the 56 weeks of antibody assessments, the efficacy of the TT-6-AmHap was lost at week 54 challenge in both the TI and TPP tests. This confirms our previously published work demonstrating that antibody titer does not correlate with efficacy against heroin challenge^38^. However, the passive infusion of monoclonal antibodies has shown to block the antinociceptive effects of fentanyl and other opioids, suggesting that antibody concentration may indeed correlate with efficacy^39–41^. Moreover, analysis of failed Phase 3 clinical trials with cocaine and nicotine indicated that a subgroup of participants in which efficacy was observed had high levels of antibodies^42,43^. More accurate methods are needed to assess the correlation of antibody concentration with efficacy^44^.

Interestingly, except for the week 15 challenge, which was before the last immunization, the antibody responses did not significantly drop after the repeated heroin challenges in the delayed dosing group. The mid-point antibody titers maintained a kinetic profile similar to the standard dosing group (Fig. 5b). Moreover, we also observed a tendency for an increase in antibody titers following the second and third heroin challenge (*p* = 0.706 and 0.0118, respectively) (Fig. 5b, Suppl. Table 3), but not after the first and fourth challenge. This could indicate a possible immune boosting by the drug, which was particularly surprising given the fact that heroin is a small molecule and is non-immunogenic. This could be incidental as the mid-point titers fluctuate between time points both in the standard and delayed dosing regimens. Another possibility for the increased titers could be the timing of antibody assessment relative to the time of challenge. Although the mechanism of clearance of the antibody-drug complex is unclear, one would assume that following a drug challenge, the entire complex is removed resulting in the decline of antibodies after challenge. Since this was not the case, one could assume that the drug is released from the antibody-drug complex and secreted in the urine resulting in the antibody returning to the circulation. However, given the high affinity of the antibodies to heroin and its metabolites (K_d_ = 0.4–2 nM), this would be a slow process^44^. Additional challenges within the next 2 days could have helped to determine if protection was restored by either old antibodies returning to the bloodstream or new antibodies being synthesized.

Taken together, the TT-6-AmHap heroin vaccine elicited robust and durable antibody responses in rodents and effectively protected the animals against heroin or drug-induced antinociception. Our findings contribute to the growing body of research on opioid conjugate vaccines, showing that flexible booster schedules do not compromise long-term immunity. By demonstrating the potential of opioid conjugate vaccines as a complimentary treatment of opioid use disorders, our results reveal that careful consideration of immunogenic and behavioral endpoints, as well as assay sensitivity, are critical for the advancement of opioid vaccine development.

## Methods

### Heroin conjugate vaccine synthesis

The heroin hapten 6-AmHap (N-((7S,7aR,12bS)-7-acetamido-3-methyl- 2,3,4,4a,5,6,7,7a-octahydro-1H-4,12-methanobenzofuro[3,2-e]isoquinolin-9-yl)-3-(tritylthio)propanamide) was coupled to the tetanus toxoid (TT) carrier protein (MassBiologics, Boston, MA) using optimized coupling procedures as described in refs. ^38,45^. Briefly, TT was incubated with the SM(PEG)_2_ cross-linker (Pierce Protein Research/Thermo Fisher Scientific, Rockford, IL) at a molar ratio of 1:1600 for 2 h. To generate conjugates with varying hapten densities, TT carrier protein was treated with increasing molar ratios of the SM-(PEG)₂ crosslinker (100, 200, 400, 800, 1600, or 3200), using aliquots containing 2 mg of protein and a 250 mM SM-(PEG)₂ stock solution in dimethyl sulfoxide. After 2 h incubation at room temperature, excess linker was removed using a spin desalting column, and protein concentration was determined via the BCA assay (Pierce Protein Research/ThermoFisher Scientific, Rockford, IL). Deprotected 6-AmHap haptens were gradually added dropwise to a continuously stirred TT-SM-(PEG)₂ intermediate solution at a molar ratio of 1:300 and incubated at room temperature for 2 h. Unconjugated haptens were removed through overnight dialysis in PBS (pH 7.4) at 4 °C. TT-6-Amhap conjugates were sterile filtered and quantified by BCA assay. The number of 6-AmHap haptens attached per TT molecule was determined using matrix-assisted laser desorption ionization time-of-flight (MALDI-TOF) mass spectrometry (MS) and protein integrity was confirmed by gel electrophoresis. Hapten densities of 30–32 haptens to TT molecules were used in all experiments except for the ones that were stated in Fig. 2.

### Adjuvants

Army Liposome Formulation (ALF43) consisted of 1,2-dimyristoyl-*sn*-glycero-3-phosphocholine (DMPC), synthetic cholesterol, 1,2-dimyristoyl-sn-glycero-3-phospho-(1’-rac-glycerol) (DMPG) at a molar ratio of 9:7.5:1, along with synthetic monophosphoryl lipid A (MPLA) (3D-PHAD^®^ from Avanti Polar Lipids, Alabaster, AL). A molar ratio of 1:8.8 was maintained between 3D-PHAD® and phospholipids, and cholesterol accounted for 43 mole percent of the formulation. ALF adjuvant was prepared by lipid deposition method as described in refs. ^19,46,47^. In short, DMPC, DMPG, cholesterol, and 3D-PHAD^®^ were solubilized in solvents and dried via rotary evaporation. Multilamellar liposomal vesicles (MLV) were generated by hydrating the lipid film with PBS (pH 7.4) to achieve the desired final phospholipid concentration. The MLV were microfluidized using a Microfluidics LV1 low volume high shear microfluidizer (Microfluidics, Westwood, MA) at 30,000 psi to form small unilamellar liposomal vesicles (SUV) as described in ref. ^47^. The SUV were lyophilized and, upon reconstitution, yielded a final phospholipid concentration of 2.29 mM for mouse study and 5.725 mM for rat study. For mouse dose, 10 µg TT-6-AmHap was added to Alhydrogel^®^ (30 µg aluminum) (Brenntag, Denmark). For rat dose, 10 – 100 µg TT-6-AmHap was added to Alhydrogel^®^ (600 µg aluminum). The mixture was gently swirled to mix for 10–60 s and incubated for 10 minutes at room temperature. PBS (pH 7.4) was added to the TT-6-AmHap-Alhydrogel^®^ mixture, then combined with lyophilized ALF. The reconstituted ALF-TT-6-AmHap-Alhydrogel^®^ mixture was vortexed (Thermo Scientific LP Vortex Mixer, Rockford, IL) for 5 minutes at 1,500 rpm at room temperature. The final vaccine formulation was 10 μg TT, 30 μg aluminum hydroxide in Alhydrogel^®^, and 20 μg 3D-PHAD^®^ in ALF per mouse dose of 50 μL in PBS, pH 7.4^19,46,47^. The final vaccine formulation for rats consisted of 10–100 μg TT, 600 μg aluminum hydroxide in Alhydrogel^®^, and 200 μg 3D-PHAD^®^ in ALF per rat dose of 200 μL in PBS, pH 7.4.

### BSA-Hapten conjugate synthesis

BSA (Pierce Protein Research/ThermoFisher Scientific, Rockford, IL) and SM-(PEG)_2_ linker (Pierce Protein Research/ThermoFisher Scientific, Rockford, IL) were incubated in PBS at a molar ratio of 1:10 for 2 h at room temperature followed by excess linker removal by spin desalting column. 6-AmHap was gradually added dropwise to a continuously stirred BSA-SM-(PEG)₂ intermediate solution at a molar ratio of 1:300. The mixture was kept at room temperature for 2 h, then dialyzed overnight at 4 °C in PBS to remove extra 6-AmHap. Following membrane filtration, the concentration of the BSA-6-AmHap conjugate was determined using the Pierce BCA assay kit (Pierce Protein Research/ThermoFisher Scientific, Rockford, IL). At a 1:10 BSA:SM-(PEG)₂ molar ratio, the conjugation efficiency yielded about 5 haptens per BSA molecule^45^.

### Animal studies experimental design

Female BALB/c mice (*n* = 10 per group) of 6–7 weeks of age (Jackson Laboratories, Bar Harbor, ME) were used. Sprague Dawley male rats from Charles River Labs (Wilmington, MA) (*n* = 10 per group) of 6 weeks of age were used. Both were randomly assigned to groups at the start of the experiments. Animals were cared for in accordance with local, state, federal, and institutional policies and were housed in the animal facility of WRAIR, which is an American Association for Accreditation of Laboratory Animal Care International (AAALAC International)-accredited facility. Studies were carried out in accordance with the recommendations in the Guide for the Care and Use of Laboratory Animals of the National Institutes of Health. The protocols were approved by the Institutional Animal Care and Use Committee at WRAIR. Standard mouse and rat experiments consisted of 4 intramuscular (IM) immunizations in alternative legs with 50 μL (for mice) and 200 μL (for rats) of the TT-6-AmHap vaccine at weeks 0, 3, 6, and 14, followed by a drug challenge at week 18. All animals were bled prior to each immunization and at week 16. Mice were bled by nicking of the tail. Phlebotomy of rats was from the tail vein with needle and syringe. For the standard versus delayed dosing studies, rats were immunized, challenged, and bled according to the protocol in Fig. 5a. Both rats and mice were anesthetized in a chamber with CO_2_ gas and then exsanguinated via cardiac puncture followed by euthanasia by cervical dislocation.

### Opioid challenges and vaccine efficacy assessments

Opioids were administered to mice by the subcutaneous route (SC) between the front shoulders. Opioids were administered to rats by intravenous (IV) tail injection. Opioid doses were formulated to give an injection volume of 0.1 mL for both mice and rats. For heroin (Lipomed, Cambridge, MA) challenge, both mice and rats were challenged at week 18 with 1 and 0.5 mg/kg heroin and tested 15 and 20 minutes, respectively after challenge dose administration. For prescription opioids challenge, mice were challenged at week 18 with 15 mg/kg morphine sulfate (Sigma-Aldrich, St Louis, MO), 4 mg/kg oxycodone hydrochloride (Sigma-Aldrich, St. Louis, MO), 4 mg/kg hydrocodone (Lipomed, Cambridge, MA), 1 mg/kg oxymorphone (Lipomed, Cambridge, MA), 1 mg/kg hydromorphone (Lipomed, Cambridge, MA). Rats were challenged with 3.5 mg/kg morphine, 3 mg/kg oxycodone, 3 mg/kg hydrocodone, 0.5 mg/kg oxymorphone, 0.5 mg/kg hydromorphone. For the repeat dose challenge, rats were injected with 0.5 mg/kg heroin every 20 minutes at weeks 10 and 15, and with 1 mg/kg heroin every 20 min at weeks 18, 24, 40, and 54. Drugs were solubilized in sterile 0.9% saline (Hospria, Lake Forest, IL). Challenge experiments were conducted by at least two staff, who confirmed animal responses in the tests below. The staff were blind to the immunization status of the animals.

### Tail-immersion test

The tail-immersion test was used for mice and rats. The temperature of the water bath was set to 54 °C. The tail was immersed in the warm water bath at 2 cm deep, and the time it took to remove the tail from the thermal stimulus was recorded. The cutoff time was set to 10 s. For rats, the tail was immersed 6 cm in the warm water.

### Hot plate test

The hot plate test was used for mice. Mice were placed in an acrylic cylinder [20 (depth) × 25 (height) cm] on a hot plate analgesia meter (Harvard Apparatus, Holliston, MA) set at 58 °C, and the latency to lift or lick the hind paw or jumping was recorded. The cutoff time was set to 30 s.

Latency data from both tail-immersion and hot plate tests were converted to percent of maximal possible effect (%MPE):

$$\% {\rm{MPE}}=[({\rm{test\; time}}-{\rm{baseline\; time}})/({\rm{cutoff\; time}}-{\rm{baseline\; time}})]\times 100.$$

High %MPE values signified a strong antinociceptive response.

### Thermal place preference test

The thermal place preference test was conducted using the BIO-T2CT thermal place preference, two-temperature zone choice Test (TPPT) with DHCP detection software (Bioseb, EB Instruments, Pinellas Park, FL) It was used, which is equipped with a infrared camera for monitoring animal movement between the plates. The plates were 165 × 165 mm each surrounded by a Plexiglas barrier. Unchallenged rats were trained for 2 days in the week before challenge with temperature one plate was set at 54 °C, and the other plate was set at 25 °C to ensure that animals had become familiar with the apparatus and rapidly move to the colder plate when placed in on the hot place of the apparatus. On the day of challenge, one plate was set at 54 °C and the other plate was set at 25 °C. Rats were gently placed on the hot side and allowed to move freely from plate to plate for 3 min. Baseline assessments were taken for each animal prior to opioid challenge. A 30-second cutoff was set to prevent burns. At the 30-second cutoff animals were removed and were scored as having spent 100% time on the hot side. The number of crossings between the 2 plates and the percentage of time spent on the hot plate were calculated using the equation: %Time in hot zone = [(time in hot zone) / (total time)] x 100.

### Antibody measurement by ELISA

The serum antibody levels against 6-AmHap were measured at week 16 or according to the protocol in Fig. 5a. 6 AmHap was coupled to BSA as described above. BSA-6-AmHap (0.1 μg BSA/0.1 mL DPBS, pH 7.4) was added to the ELISA plates (Nunc MaxiSorp, ThermoFisher Scientific, Rockford, IL) and incubated overnight at 4 °C. The remainder of the ELISA was conducted the following day as described in ref. ^28^. Briefly, the plates were blocked with blocker (1% BSA in 20 mM Tris-0.15 M sodium chloride, pH 7.4 for mice or 0.5% milk and 0.1% Tween-20 in 1xDPBS pH 7.4 for rats), for 2 h. Mouse or rat sera were serially diluted 2-fold at 1:400 in blocker and added to the plates in triplicates. Following incubation for 1 h at room temperature, the plates were washed with 20 mM Tris-0.15 M sodium chloride-0.1% Tween-20. Plates were then incubated for another hour at room temperature with peroxidase-linked sheep anti-mouse IgG HRP (The Binding Site, San Diego, CA) at 1:1000 in blocker for mice, or with goat anti-rat IgG-Fc Fragment antibody HRP conjugated (Bethyl, Montgomery, TX) at 1:1000 in blocker for rats. After washing off the secondary antibodies, 100 μL/well KPL TMB 2-component microwell peroxidase substrate system (SeraCare Life Sciences, Oceanside, CA) was added to plates for 1 hour, followed by the addition of 100 μL/well stopping solution, 1% SDS (Sigma-Aldrich, St. Louis, MO). The absorbance was determined at 405 nm. Binding antibody response is presented as mid-point titer. The mid-point titers represent the dilution at which half of the vaccine’s maximum effect was observed, which is less prone to variability compared to end-point titers.

### Data analysis

Computational and statistical analysis was performed using GraphPad Prism 10.5.0 (GraphPad Software, La Jolla, CA, USA). All values are reported as mean ± SEM. Antibody titers were expressed as serum dilution at the midpoint of the dilution curve, as interpolated from non-linear fit, [Inhibitor] vs. response, Variable slope (four parameters) least squares fit. Midpoint titers represented the IC_50_ value of best-fit-value. Data were analyzed using one-way ANOVA or as multiple unpaired t-tests, as indicated in the figure legends. Tukey’s multiple comparisons test was conducted as a post hoc analysis to determine significant differences among groups. Comparison of antibody titers over time between standard and delayed dosing regimen groups used a mixed-effects analysis with Geisser-Greenhouse correction and Tukey’s multiple comparisons test, with individual variances computed for each comparison. Comparison of TT-6-AmHap efficacy against repeated heroin challenges between standard and delayed dosing regimen groups used a mixed-effects model, where EC_50_ were calculated using non-linear regression, [Agonist] vs. response, Variable slope (four parameters) analysis. The EC_50_ for the TPP tests on Weeks 10, 40, and 54 used linear regression to calculate the y-intercept (b) and slope (m). These dose curves were linear and did not meet the criteria for using the four-parament analysis. The EC_50_ was calculated using the equation for a straight line: y = mx + b, where y was set at 50% and the equation was solved for x, which is the EC_50_.

### Ethical approval

The experiments reported herein were conducted in compliance with the Animal Welfare Act and per the principles set forth in the “Guide for Care and Use of Laboratory Animals,” Institute of Laboratory Animals Resources, National Research Council, National Academy Press, 2011. The work was conducted under a WRAIR IACUC-approved protocol.

## Supplementary information

Supplementary Material - NPJ Vaccines EK_GM_Final

## Data Availability

All data associated with this study are present in the paper or in the Supplementary Material Section. The raw data sets generated during and/or analyzed during the current study are available from the corresponding author upon request.
