# Supplementary material for: Optimization of heroin conjugate vaccine performance in rodent models
Source: NPJ Vaccines. 2026 Jul 14;11:206. doi: 10.1038/s41541-026-01517-6 (PMC13736862; doi:10.1038/s41541-026-01517-6)
Supplement: Supplementary file 1 [file 41541_2026_1517_MOESM1_ESM.docx]

**Supplementary Material**

**Optimization of Heroin Conjugate Vaccine Performance in Rodent Models**

Essie Komla, Connor Whalen, Oscar B. Torres, Agnieszka Sulima, Arthur E. Jacobson, Kenner C. Rice, and Gary R. Matyas

Supplemental Figure 1. Dose response curves of opioids injected by the subcutaneous route in mice. Female Balb/c mice were injected at the dose indicated in approximately 0.1 mL of saline by the subcutaneous route on the back between the front shoulder blades. Animals were assayed in the hot plate and tail flick assays as described in the methods.

Supplemental Figure 2. Dose response curves of opioids injected by the intravenous route in rats. Male Sprague-Dawley rats were injected at the dose indicated in approximately 0.1 mL of saline by in the tail vein near the base of the tail. Animals were assayed in the hot plate (heroin only) and tail immersion assays as described in the methods.

**Supplementary Table 1. Mid-point antibody titers to 6‑AmHap in rats comparing the standard verses delayed dosing regimen**

| **Time**  **(Weeks)** | **Standard dosing regimen** | | **Delayed dosing regimen** | | **Significance**  **(p)*** |
| --- | --- | --- | --- | --- | --- |
|  | **Mean** | **SE** | **Mean** | **SE** |  |
| 0 | 10^†^ | 0 | 10^†^ | 0 | NA^⁑^ |
| 3 | 123,239 | 15,995 | 136,246 | 22,460 | >0.99 |
| 6 | 294,712 | 40,650 | 369,646 | 58,533 | >0.99 |
| 8 | 491,824 | 51,671 | 288,338 | 37,125 | 0.37 |
| 11 | ND^‡^ | ND | 179,221 | 24,049 | NA^⁑^ |
| 13 | ND | ND | 387,926 | 51,494 | NA^⁑^ |
| 14 | 151,145 | 17,625 | ND | ND | NA^⁑^ |
| 16 | 390,973 | 34,872 | ND | ND | NA^⁑^ |
| 19 | 221,973 | 24,310 | 240,058 | 32,390 | >0.99 |
| 21 | 198,587 | 21,550 | 353,324 | 37,322 | 0.1435 |
| 24 | 163,950 | 18,966 | 588,456 | 82,049 | 0.0071 |
| 31 | 278,281 | 31,597 | 625,942 | 83,474 | 0.2175 |
| 34 | 256,386 | 26,290 | 485,768 | 84,354 | 0.504 |
| 37 | 120,283 | 11,177 | 232,313 | 35,601 | 0.5754 |
| 40 | 245,581 | 67,719 | ND | ND | NA^⁑^ |
| 44 | 212,897 | 72,811 | 383,079 | 36,891 | >0.99 |
| 52 | 871,77 | 13,307 | 219,890 | 31,433 | 0.641 |
| 56 | 64,789 | 7,366 | 159,627 | 31,888 | 0.7183 |

^*^p values were determined using GraphPad Prism Mixed Effects Analysis with Geisser-Greenhouse correction and Turkey’s correction for multiple comparisons.

^†^Antibodies to 6-AmHap were not detected in the pre-immune sera samples. Value 10 was assigned to allow for statistical analysis.

^⁑^NA – non applicable

^‡^ND – not done; Sera samples were not taken at these data points

**Supplemental Table 2. Statistical Comparison of mid-point antibody titers to 6‑AmHap in rats over time within the dosing regimen**

| **Time points analyzed*** | | **Standard dosing regimen** | **Delayed dosing regimen** |
| --- | --- | --- | --- |
| **Time**  **(weeks)** | **Time**  **(weeks)** | **Significance**  **(p)** | **Significance**  **(p)*** |
| 3 | 6 | 0.051 | 0.0396 |
|  | 8 | 0.0009 | 0.0154 |
|  | 11 | ND^†^ | 0.694 |
|  | 13 | ND | 0.009 |
|  | 14 | 0.6022 | ND |
|  | 16 | 0.0008 | ND |
|  | 19 | 0.0631 | 0.0696 |
|  | 21 | 0.2745 | 0.0068 |
|  | 24 | 0.9366 | 0.0273 |
|  | 31 | 0.0516 | 0.0103 |
|  | 34 | 0.0287 | 0.1936 |
|  | 37 | >0.9999 | 0.4666 |
|  | 40 | 0.9742 | ND |
|  | 44 | 0.9997 | 0.0214 |
|  | 52 | 0.8051 | 0.9841 |
|  | 56 | 0.5683 | >0.9999 |
| 6 | 8 | 0.0307 | 0.5917 |
|  | 11 | ND | 0.0599 |
|  | 13 | ND | >0.9999 |
|  | 14 | 0.135 | ND |
|  | 16 | 0.8942 | ND |
|  | 19 | 0.8744 | 0.5657 |
|  | 21 | 0.6684 | >0.9999 |
|  | 24 | 0.4339 | 0.3703 |
|  | 31 | >0.9999 | 0.3744 |
|  | 34 | >0.9999 | 0.9998 |
|  | 37 | 0.1127 | 0.6987 |
|  | 40 | >0.9999 | ND |
|  | 44 | >0.9999 | 0.9996 |
|  | 52 | 0.2147 | 0.5391 |
|  | 56 | 0.1784 | 0.9315 |
| 8  8 | 11 | ND | 0.0298 |
|  | 13 | ND | 0.7849 |
|  | 14 | 0.0008 | ND |
|  | 16 | 0.7566 | ND |
|  | 19 | 0.0168 | 0.9758 |
|  | 21 | 0.0649 | 0.9633 |
|  | 24 | 0.0131 | 0.1494 |
|  | 34 | 0.0338 | 0.8581 |
|  | 37 | 0.0038 | 0.9859 |
|  | 40 | 0.3983 | NA |
|  | 44 | 0.285 | 0.9856 |
|  | 52 | 0.0119 | 0.6624 |
|  | 56 | 0.0487 | 0.995 |
| 11 | 13 | ND | 0.029 |
|  | 14 | ND | ND |
|  | 16 | ND | ND |
|  | 19 | ND | 0.6776 |
|  | 21 | ND | 0.0143 |
|  | 24 | ND | 0.0285 |
|  | 31 | ND | 0.0207 |
|  | 34 | ND | 0.2937 |
|  | 37 | ND | 0.9777 |
|  | 40 | ND | ND |
|  | 44 | ND | 0.1043 |
|  | 52 | ND | >0.9999 |
|  | 56 | ND | >0.9999 |
| 13 | 14 | ND | ND |
|  | 16 | ND | ND |
|  | 19 | ND | 0.0583 |
|  | 21 | ND | >0.9999 |
|  | 24 | ND | 0.8562 |
|  | 31 | ND | 0.1899 |
|  | 34 | ND | 0.9994 |
|  | 37 | ND | 0.1061 |
|  | 40 | ND | ND |
|  | 44 | ND | >0.9999 |
|  | 52 | ND | 0.305 |
|  | 56 | ND | 0.5393 |
| 14 | 16 | 0.0011 | ND |
|  | 19 | 0.2768 | ND |
|  | 21 | 0.7836 | ND |
|  | 24 | >0.9999 | ND |
|  | 31 | 0.0787 | ND |
|  | 34 | 0.0726 | ND |
|  | 37 | 0.1287 | ND |
|  | 40 | 0.9977 | ND |
|  | 44 | >0.9999 | ND |
|  | 52 | 0.2061 | ND |
|  | 56 | 0.5382 | ND |
| 16  16 | 19 | 0.0015 | ND |
|  | 21 | 0.0026 | ND |
|  | 24 | 0.0005 | ND |
|  | 31 | 0.0123 | ND |
|  | 34 | 0.0027 | ND |
|  | 37 | 0.0017 | ND |
|  | 40 | 0.5891 | ND |
|  | 44 | 0.3499 | ND |
|  | 52 | 0.0017 | ND |
|  | 56 | 0.017 | ND |
| 19 | 21 | 0.5581 | 0.1078 |
|  | 24 | 0.0263 | 0.1124 |
|  | 31 | 0.2966 | 0.0131 |
|  | 34 | 0.4236 | 0.4432 |
|  | 37 | 0.02 | >0.9999 |
|  | 40 | >0.9999 | ND |
|  | 44 | >0.9999 | 0.8092 |
|  | 52 | 0.016 | 0.8972 |
|  | 56 | 0.1278 | 0.1895 |
| 21 | 24 | 0.0497 | 0.3746 |
|  | 31 | 0.0272 | 0.1777 |
|  | 34 | 0.1033 | 0.9942 |
|  | 37 | 0.0372 | 0.0907 |
|  | 40 | >0.9999 | ND |
|  | 44 | >0.9999 | >0.9999 |
|  | 52 | 0.0143 | 0.2546 |
|  | 56 | 0.1352 | 0.3475 |
| 24 | 31 | 0.0026 | >0.9999 |
|  | 34 | 0.0021 | >0.9999 |
|  | 37 | 0.2821 | 0.0973 |
|  | 40 | 0.9941 | ND |
|  | 44 | >0.9999 | 0.3784 |
|  | 52 | 0.0122 | 0.1464 |
|  | 56 | 0.1836 | 0.2911 |
| 31 | 34 | 0.9879 | 0.9803 |
|  | 37 | 0.0115 | 0.0038 |
|  | 40 | >0.9999 | ND |
|  | 44 | 0.9985 | 0.1348 |
|  | 52 | 0.0062 | 0.0038 |
|  | 56 | 0.0332 | 0.4887 |
| 34 | 37 | 0.0061 | 0.3589 |
|  | 40 | >0.9999 | ND |
|  | 44 | >0.9999 | 0.9976 |
|  | 52 | 0.0021 | 0.1963 |
|  | 56 | 0.0432 | 0.4274 |
| 37 | 40 | 0.9207 | ND |
|  | 44 | 0.9977 | 0.0118 |
|  | 52 | 0.3172 | 0.3255 |
|  | 56 | 0.6824 | 0.9944 |
| 40 | 44 | 0.8501 | ND |
|  | 52 | 0.6451 | ND |
|  | 56 | 0.0478 | ND |
| 44 | 52 | 0.9395 | 0.4516 |
|  | 56 | 0.1082 | 0.6969 |
| 52 | 56 | >0.9999 | 0.9993 |

^*^p values were determined using GraphPad Prism Mixed Effects Analysis with Geisser-Greenhouse correction and Turkey’s correction for multiple comparisons.

^†^ND – Not done; Sera samples were not taken at these data points

Supplementary Table 3. Comparison of antibody titers before and after heroin challenge in rats immunized with TT‑6‑AmHap using the delayed dosing regimen.

| **Challenge**  **(Week)** | **Mid-point titer before challenge**  **(mean ± SEM)** | **Mid-point titer after challenge**  **(mean ± SEM)** | **Significance**  **(p)*** |
| --- | --- | --- | --- |
| 15 | Week 13: 387,926 ± 51,494 | Week 19: 240,058 ± 32,039 | 0.001 |
| 24.5 | Week 24: 588,456 ± 84,029 | Week 31: 625,942 ± 83,474 | 0.706 |
| 40 | Week 37: 232,313 ± 35,601 | Week 44: 383,079 ± 36,891 | 0.0118 |
| 54 | Week 52: 219,890 ± 31,433 | Week 56: 159,627 ± 31,888 | 0.2199 |

^*^Titers between the two time points before and after challenge were compared using paired T-test.
